# Supplementary material for: Ginsenoside Rg3 Induces Browning of 3T3-L1 Adipocytes by Activating AMPK Signaling
Source: Nutrients. 2020 Feb 7;12(2):427. doi: 10.3390/nu12020427 (PMC7071202; doi:10.3390/nu12020427)
Supplement: Supplementary file 1 [file nutrients-12-00427-s001.zip › nutrients-693950-supplementary.pptx]

## Slide 1
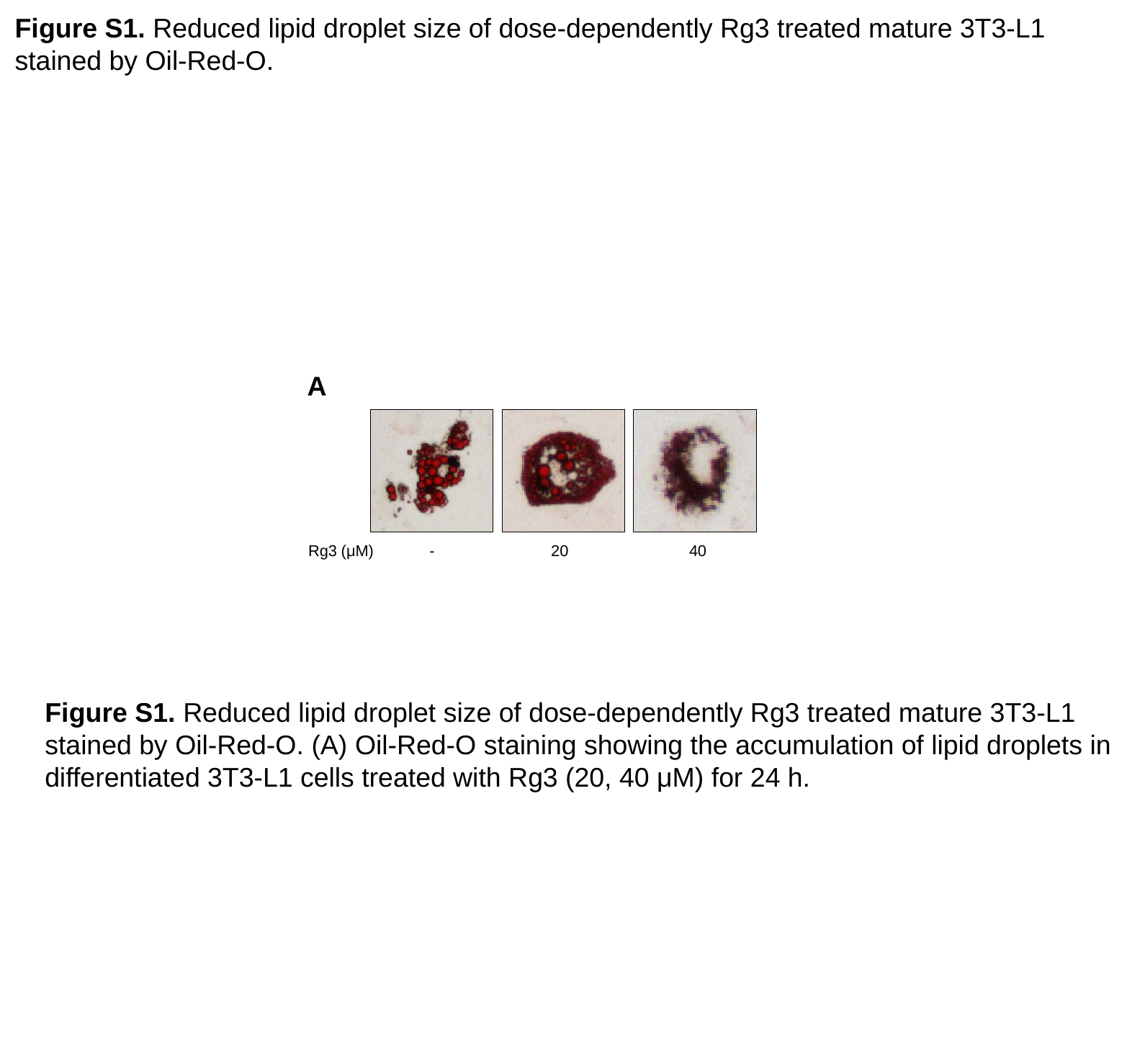

Figure S1. Reduced lipid droplet size of dose-dependently Rg3 treated mature 3T3-L1 stained by Oil-Red-O.
A
Rg3 (μM)
 - 20 40
Figure S1. Reduced lipid droplet size of dose-dependently Rg3 treated mature 3T3-L1 stained by Oil-Red-O. (A) Oil-Red-O staining showing the accumulation of lipid droplets in differentiated 3T3-L1 cells treated with Rg3 (20, 40 μM) for 24 h.

## Slide 2
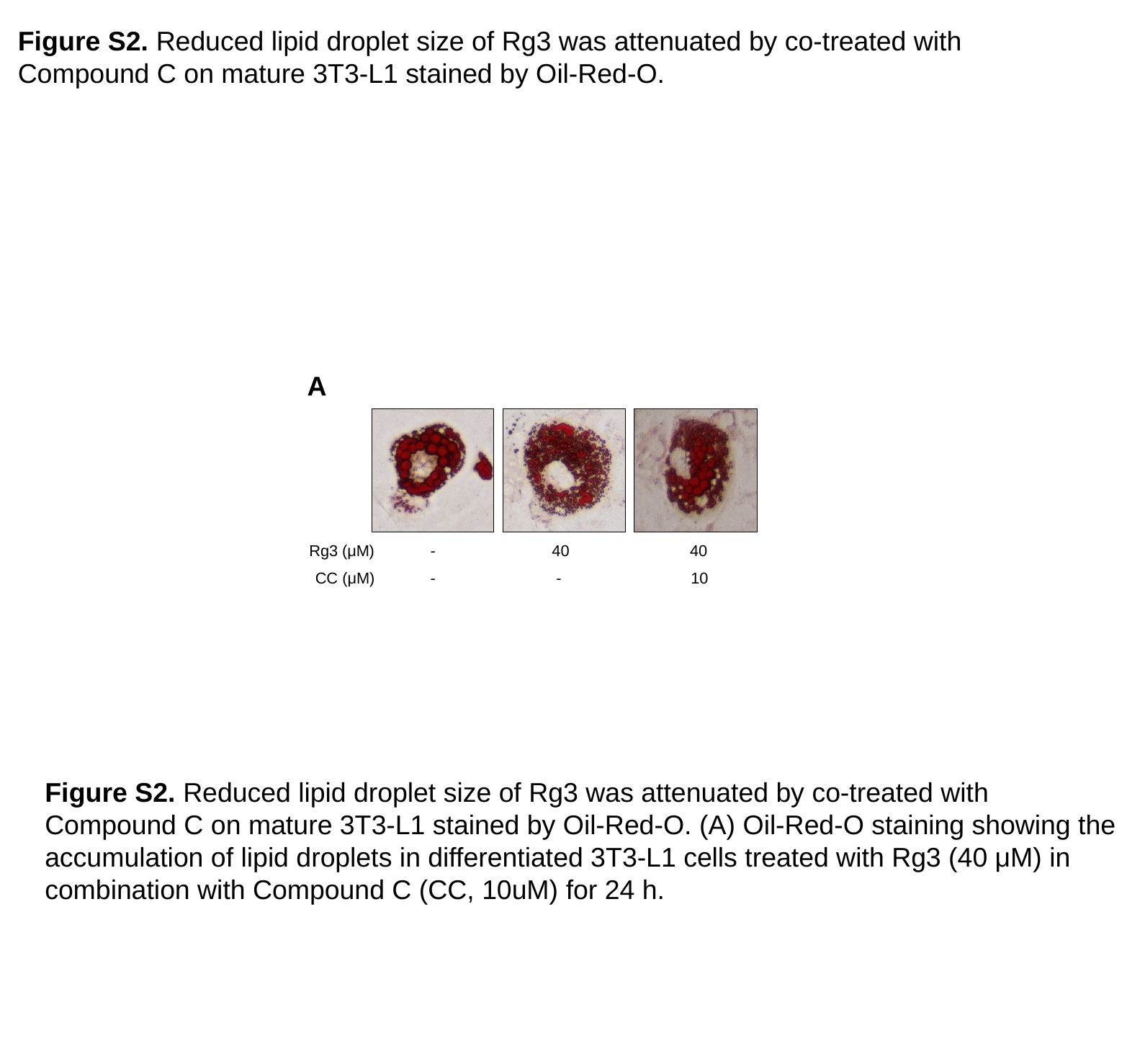

Figure S2. Reduced lipid droplet size of Rg3 was attenuated by co-treated with Compound C on mature 3T3-L1 stained by Oil-Red-O.
A
Rg3 (μM)
 - 40 40
CC (μM)
 - - 10
Figure S2. Reduced lipid droplet size of Rg3 was attenuated by co-treated with Compound C on mature 3T3-L1 stained by Oil-Red-O. (A) Oil-Red-O staining showing the accumulation of lipid droplets in differentiated 3T3-L1 cells treated with Rg3 (40 μM) in combination with Compound C (CC, 10uM) for 24 h.
